# ROS-Mediated Cytotoxic Effect of Copper(II) Hydrazone Complexes against Human Glioma Cells

**DOI:** 10.3390/molecules191117202

**Published:** 2014-10-27

**Authors:** Angel A. Recio Despaigne, Jeferson G. Da Silva, Pryscila R. da Costa, Raquel G. dos Santos, Heloisa Beraldo

**Affiliations:** 1 Departamento de Química, Universidade Federal de Minas Gerais, 31270-901 Belo Horizonte, Brazil; E-Mails: areciod@yahoo.com (A.A.R.D.); jefersonpi1@yahoo.com.br (J.G.D.S.); 2 Departamento de Farmácia, Universidade Federal de Juiz de Fora (UFJF), Campus Governador Valadares, Av. Dr. Raimundo Monteiro de Rezende, 330, Centro, 35010-177 Governador Valadares, Brazil; 3 Centro de Desenvolvimento da Tecnologia Nuclear (CDTN), 31270-901 Belo Horizonte, Brazil; E-Mails: pryrodriigues@yahoo.com.br (P.R.C.); raqueou@uol.com.br (R.G.S.)

**Keywords:** pyridine-derived hydrazones, copper(II) complexes, cytotoxicity, glioma cells, ROS generation

## Abstract

2-Acetylpyridine acetylhydrazone (H2AcMe), 2-benzoylpyridine acetylhydrazone (H2BzMe) and complexes [Cu(H2AcMe)Cl_2_] (**1**) and [Cu(H2BzMe)Cl_2_] (**2**) were assayed for their cytotoxicity against wild type p53 U87 and mutant p53 T98 glioma cells, and against MRC-5 fibroblast cells. Compounds **1** and **2** proved to be more active than the corresponding hydrazones against U87, but not against T98 cells. Compound **1** induced higher levels of ROS than H2AcMe in both glioma cell lines. H2AcMe and **1** induced lower levels of ROS in MRC5 than in U87 cells. Compound **2** induced lower levels of ROS in MRC5 than in T98 cells. The cytotoxic effect of **1** in U87 cells could be related to its ability to provoke the release of ROS, suggesting that the cytotoxicity of **1** might be somehow p53 dependent.

## 1. Introduction

[*cis*(Diaminedichloro)platinum(II)], “cisplatin” is one of the most active anticancer drugs, but its use is limited by undesirable side-effects and the appearance of cellular resistance. The mode of action of cisplatin involves covalent DNA binding, hence the search for more effective target-specific non-covalently DNA binding anticancer drug candidates is of great interest [1]. Considerable effort has been devoted to the development of copper-based anticancer agents that can bind to and cleave DNA under physiological conditions [1].

Copper is a component of numerous enzymes and participates in scavenging free radicals, as in the case of copper-zinc superoxide dismutases [2]. Inside cells, free copper ions can go from one redox state to another under physiological conditions. This redox cycling is responsible for both the catalytic and the toxic potential of copper. Cu(II) directly interacts with several biological molecules and promotes their oxidation in a redox reaction in which Cu(I) is formed. In turn, Cu(I) reacts with molecular oxygen to form the superoxide anion O_2_, which dismutates to H_2_O_2_ and O_2_, ultimately producing hydroxyl radicals and other reactive oxygen species (ROS). ROS generation can cause oxidative DNA damage, is responsible for the clastogenic potential of copper [3,4] and can result in cell death [5].

The tumor suppressor protein p53 is a transcription factor which regulates cell cycle progression, cell survival, and DNA repair in cells exposed to genotoxic stress. Exposure to DNA damage induces p53 to accumulate in the nucleus, to bind to specific DNA sequences, and to activate several genes including effectors of the cell cycle. In many cell types, activation of p53 results in transient cell cycle arrest. In other cells, accumulation of p53 triggers apoptosis. Both processes suppress the proliferation of cells that have undergone DNA damage and contribute to prevent the propagation of cells with potentially oncogenic mutations. It has been shown that the intracellular levels and the redox activity of copper are critical for p53 protein conformation and DNA-binding activity suggesting that copper ions may participate in the physiological control of p53 function [3].

Hydrazones and their metal complexes constitute an important class of compounds with a wide range of pharmacological applications as antiviral, antimicrobial, anti-inflammatory [6] and cytotoxic agents [7]. The bioactivities of copper complexes with a variety of hydrazones have been investigated. These complexes proved to present cytotoxic [8] and antimicrobial [9] activities. It has been shown that a number of copper(II) complexes with hydrazones bind significantly to and are able to cleave DNA [10,11,12].

We previously prepared a family of copper(II) complexes with 2-acetylpyridine- and 2-bezoylpyridine-derived hydrazones [9]. In the present work [dichloro(2-acetylpyridineacetyl-hydrazone)copper(II)], [Cu(H2AcMe)Cl_2_] (**1**) and [dichloro(2-benzoylpyridineacetylhydrazone)copper(II)], [Cu(H2BzMe)Cl_2_] (**2**) were studied for their cytotoxic activities against U87 (p53 wild-type glioblastoma multiforme) and T98 (p53 mutant glioblastoma multiforme) tumor cells. The interactions of complexes **1** and **2** with DNA and bovinum serum albumin (BSA) were investigated as well as the role of ROS generation in the mechanism of their cytotoxic effect.

## 2. Results and Discussion

**Scheme 1:**
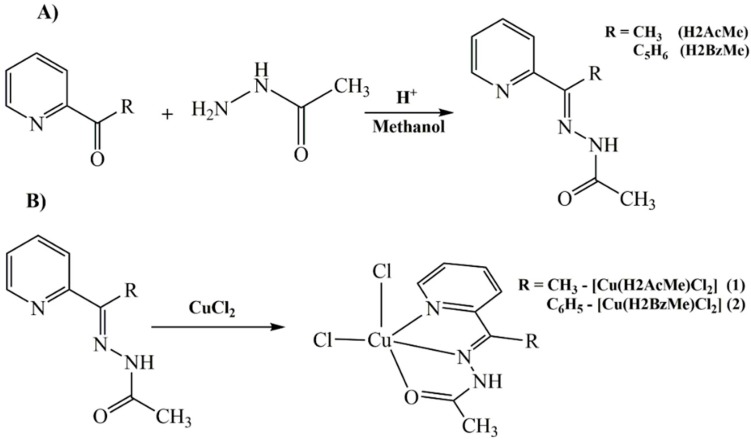
Schematicrepresentation of the syntheses of the hydrazones (**A**), and their copper(II) complexes **1** and **2** (**B**).

2-Acetylpyridine acetylhydrazone (H2AcMe), 2-benzoylpyridine acetylhydrazone (H2BzMe) and complexes **1** and **2** were prepared as previously reported by some of us [13] and by other authors [14,15].The synthetic routes for the ligands and their Cu(II) complexes are outlined in Scheme 1.

### 2.1. Cytotoxic Activity against Malignant U-87 and T-98 Glioma Cells and against MRC5 Cells

The *in vitro* cytotoxic effects of H2AcMe, H2BzMe and complexes **1** and **2** against wild type p53 U87 and mutant p53 T98 glioblastoma cells and against MRC-5 fibroblasts cells were tested using cisplatin as a positive control. To determine the IC_50_ (concentration that inhibits 50% of cell survival) values the cytotoxic effects were quantified using the 3-(4,5-dimethyl-2-thioazolyl)-2,5-diphenyl tetrazolium bromide (MTT) colorimetric assay [16]. All tested compounds were cytotoxic against both glioma cell lines in a dose-dependent way. The IC_50_ values (see Table 1) were found in the micromolar range. Despite their cytotoxic activity, all compounds were less toxic to fibroblasts cells than cisplatin. CuCl_2_ evoked only mild cytotoxicity in all cell lineages with IC_50_ higher than 100 µM.

**Table 1:** Cytotoxic effect of 2-acetylpyridine acetylhydrazone (H2AcMe), 2-benzoylpyridine acetylhydrazone (H2BzMe) and their copper(II) complexes **1** and **2**.

Compounds	IC_50_ [µM]		
	U87	T98	MRC5
H2AcMe	67.6 ± 4.08	13.80 ± 4.89	19.80 ± 4.15
[Cu(H2AcMe)Cl_2_] (**1**)	3.02 ± 0.85	5.08 ± 0.41	22.50 ± 3.13
H2BzMe	52.30 ± 10.03	19.70 ± 1.06	62.30 ± 7.76
[Cu(H2BzMe)Cl_2_] (**2**)	5.95 ± 0.74	29.40 ± 7.06	49.40 ± 8.83
CuCl_2_	>100	>100	>100
Cisplatin	1.76 ± 0.22	5.31 ± 1.94	5.05 ± 0.71

H2AcMe and H2BzMe were significantly more potent against p53 mutant T98 cells (IC_50_ = 13.80 and 19.80 µM, respectively) than against p53 wild-type U87 cells (IC_50_ = 67.60 and 52.30 µM, respectively) (*p* < 0.05). Complexes **1** and **2** proved to be more active than the corresponding hydrazones against U87 cells (*p* < 0.001 and *p* < 0.01, respectively). Although complex **1** seemed to be more active than its ligand H2AcMe against T98 cells, this effect was not statistically significant (*p* > 0.05). Coordination to Cu(II) did not result in activity improvement against T98 cells in both complexes (see Table 1).

The hydrazones presented low selectivity index (SI = IC_50MRC5_/IC_50tumor cell_) in U87 cells. Only H2BzMe presented a good selectivity index in T98 cells (*p* < 0.05). In contrast, both complexes presented good selectivity indexes against the two glioma cell lineages (*p* < 0.05). For complex (**1**) SI = 7.45 (U87); SI = 4.42 (T98); for complex (**2**) SI = 8.30 (U87); SI = 1.68 (T98) and for cisplatin SI = 2.87 (U87); SI = 0.95 (T98).

### 2.2. Analysis of Morphological Changes

**Figure 1:**
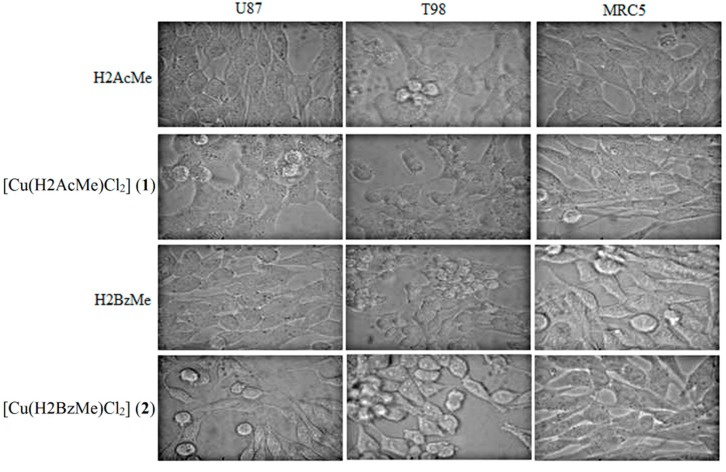
Morphological changes induced by 2-acetylpyridine acetylhydrazone (H2AcMe), 2-benzoylpyridine acetylhydrazone (H2BzMe) and their copper(II) complexes **1** and **2**. Photomicrographs of human glioblastoma cells: U-87 (p53 wild type), T-98 (expressing p53 mutant) and human fetal lung fibroblast cells, MRC5. Changes such as cell rounding, cell shrinkage and blebs formation suggest the induction of programmed cell death (magnification ×400).

Morphological alterations in the cells are shown in Figure 1. It can be observed that the treatment with test compounds induced morphological alterations such as retraction of cytoplasmatic expansions, detachment, formation of round shaped cells, cell shrinkage and membrane blebs formation.

All of these morphological changes are associated to cell death.

### 2.3. Analysis of Chromosomal DNA Alterations

**Figure 2:**
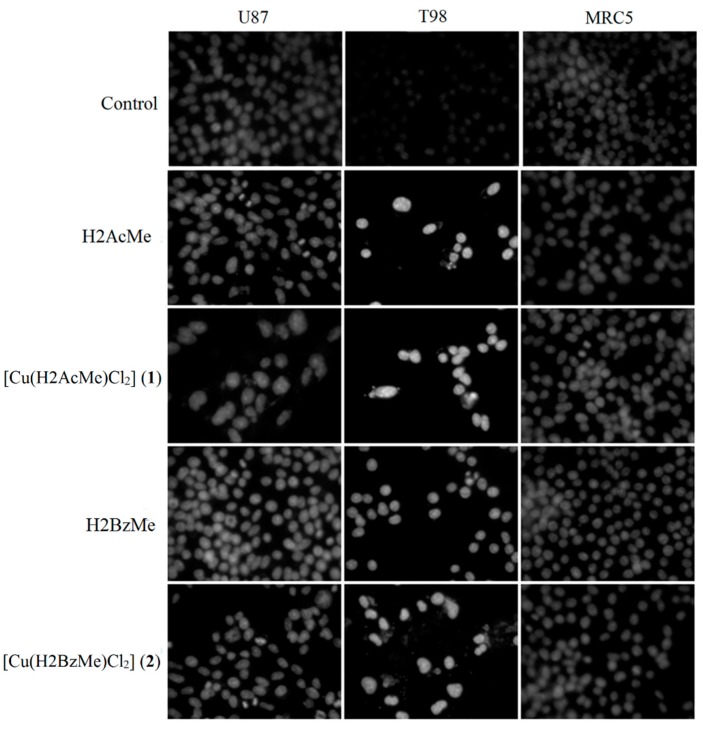
Nuclear changes induced by treatment with 2-acetylpyridine acetylhydrazone (H2AcMe), 2-benzoylpyridine acetylhydrazone (H2BzMe) and their copper(II) complexes **1** and **2**. Cells were treated with 1 × 10^−5^ mol/L of the compounds or diluent (control) and stained with DAPI. Treated cells show nuclear condensation and apoptotic bodies visualized as small focal bright points (magnification ×400).

A number of anticancer drugs exert their effects by inducing apoptosis [17]. Cytotoxic compounds that disrupt DNA and chromosomal integrity leading to apoptotic death evoke DNA condensation and fragmentation that can be visualized as very small focal bright points when stained by DAPI [18]. Analysis of chromosomal alterations were made using 4',6-diamidine-2'-phenindole dihydrocloride (DAPI) [19] (Figure 2). Unlike the untreated control cells both U87 and T98 glioma cells treated with the test compounds showed chromatin condensation and apoptotic bodies. The non-malignant fibroblast cells presented no significant chromosomal damage.

### 2.4. Measurement of Reactive Oxygen Species (ROS) Generation

**Figure 3:**
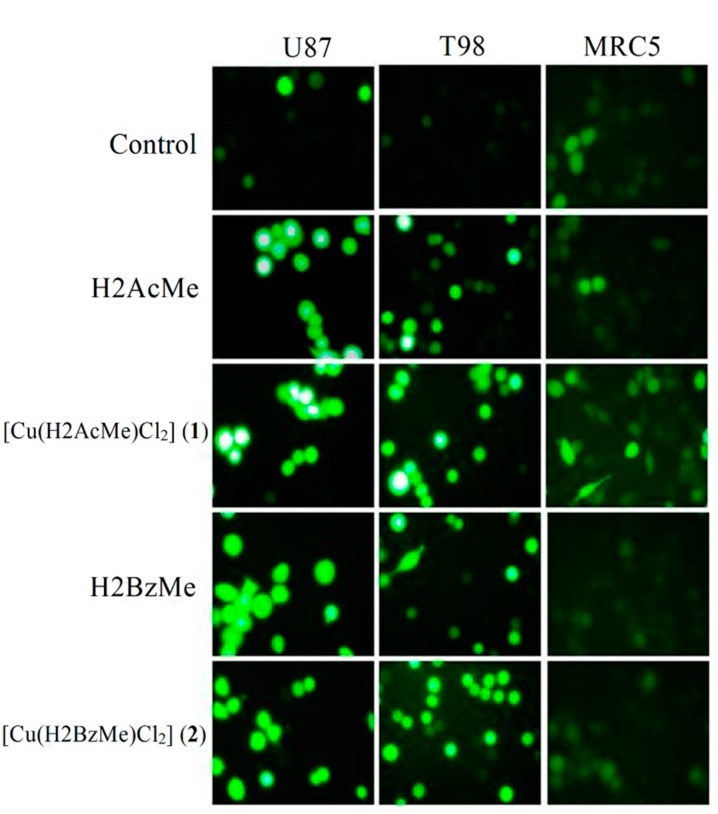
Generation of reactive oxygen species (ROS) in cells treated with 2-acetylpyridine acetylhydrazone (H2AcMe), 2-benzoylpyridine acetylhydrazone (H2BzMe) and their copper(II) complexes (**1**) and (**2**). Cells were treated for 24 h with 1 × 10^−5^ mol/L of compounds and stained with DCF-DA. Treated cells show increased fluorescence intensity (magnification ×400).

**Figure 4:**
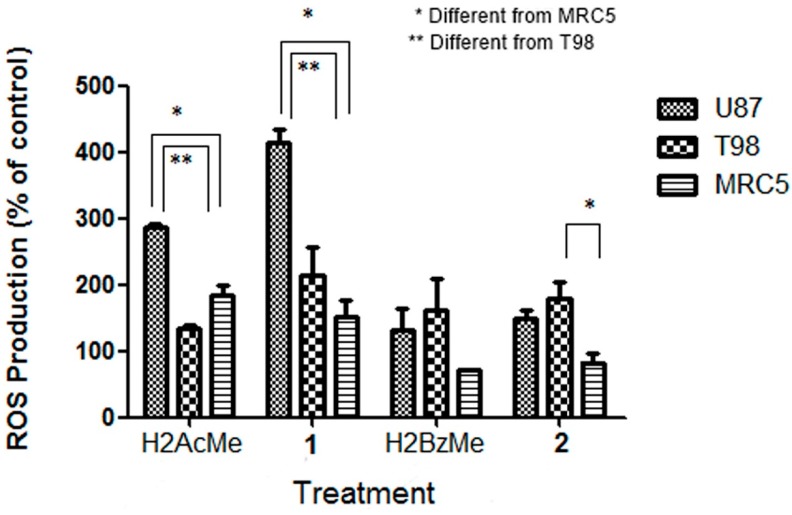
ROS production (measured as relative fluorescence increase) in cells treated with 2-acetylpyridine acetylhydrazone (H2AcMe), 2-benzoylpyridine acetylhydrazone (H2BzMe) and their copper(II) complexes **1** and **2** at 1 × 10^−5^ M.

To examine the effects of H2AcMe, H2BzMe and their complexes **1** and **2** on the generation of free radicals (reactive oxygen species, ROS), we used the 2',7'-dichlorodihydrofluorescein diacetate (DCFH-DA) assay [20]. As shown in Figure 3 and Figure 4, treatment of the glioma cells with all compounds for 24 h resulted in marked increase of the intracellular ROS levels.

H2AcMe and its complex **1** induced higher levels of ROS in U87 cells (*p* < 0.05) than in T98 cells. H2BzMe and **2** proved to be equally effective in both cell lineages (*p* > 0.05). Upon coordination of H2AcMe to Cu(II) higher levels of ROS were observed for complex **1** in U87 cells (*p* < 0.05). H2BzMe and **2** showed a similar effect on ROS generation in both malignant cell lineages (*p* > 0.05).

Interestingly, H2AcMe and its complex **1** induced lower levels of ROS in MRC5 cells (*p* < 0.05) than in U87 glioma cells. H2BzMe induced the same increasing in ROS levels in MRC5 and in both malignant cells (*p* > 0.05). Complex **2** exhibited similar effects on U87 and T98 glioma cells (*p* > 0.05). However, **2** induced lower levels of ROS in MRC5 than in T98 glioma cells (*p* < 0.05).

Several studies have implicated mitochondria-derived ROS in cell death induced by p53 [21,22]. It has also been revealed that ROS act as both an upstream signal that triggers p53 activation and a downstream factor that mediates apoptosis [23]. Sawada and colleagues have proposed two separate signaling cascades, ROS-mediated-p53-dependent and ROS-mediated-p53-independent pathways, both of which contribute to apoptosis of human glioma cells [24]. Taking together the foregoing results show that the cytotoxicity induced by the compounds under study is mediated by ROS.

In agreement with the important role of ROS in cell death induced by p53 and corroborating MTT data, complex **1** proved to be more potent against wild-type p53 U87 cells and more effective in promoting ROS generation in this cell lineage (*p* < 0.05). The p53 tumor suppressor is a pivotal regulating component that senses various intrinsic and extrinsic stresses and initiates apoptotic cell death [25]. Although the detailed mechanisms underlying the effects of the hydrazones and their copper complexes on the down-regulating p53 levels warrant further research, the present results suggest that complex **1** might induce p53-dependent apoptotic pathways mediated by ROS.

### 2.5. DNA Binding Studies

Complexes can bind to DNA *via* both covalent and/or non-covalent (intercalation, electrostatic or groove binding) interactions. In covalent binding the labile ligand of the complexes is replaced by a nitrogen atom from a DNA base such as guanine N7. Non-covalent DNA interactions include intercalative, electrostatic and groove (surface) binding. Intercalation involves the partial insertion of aromatic heterocyclic rings of the ligands between the DNA base pairs [26]. The spectroscopic techniques of electronic absorption and fluorescence are two of the most useful for DNA-binding studies of small molecules [27].

#### 2.5.1. Absorption Spectroscopy Studies of DNA Binding

The electronic absorption spectra of free complexes **1** and **2** in Tris-HCl buffer consist of two well resolved bands in the range from 250 to 400 nm. The absorption that appears at 273 nm in **1** and 279 nm in **2** is characteristic of π→π* transitions of aromatic rings. The band that was found at 341 nm in **1** and 353 nm in **2** is attributed to n→π* transitions from the ligands overlapped with ligand-to-metal charge transfer (LMCT) transitions [28].

**Figure 5:**
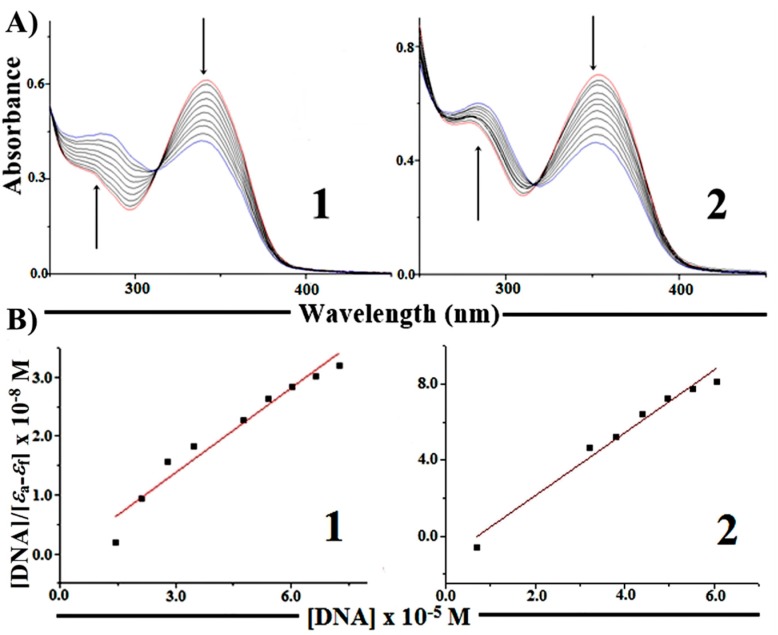
(**A**) Electronic absorption spectra of complexes (**1**) (7.25 µM) and (**2**) (6.78 µM) in Tris-HCl buffer (pH = 7.20) in the absence and in the presence of increasing amounts of CT-DNA (ten different complex: DNA molar ratios ranging from 1:1 to 1:10). Arrows show the changes in absorbance with increasing DNA concentration. (**B**) Plot of [DNA]/[ε_a_ − ε_f_] *vs.* [DNA].

The absorption spectra of **1** and **2** in the absence and in the presence of CT-DNA (at constant concentration of the complex) are shown in Figure 5.

With the increasing of CT-DNA concentration, a small shift was observed for the absorptions at 342 and 353 nm in the spectra of **1** and **2**, respectively, with a strong decrease in intensity. In addition, the bands at 273 nm for **1** and 279 nm for **2** underwent a considerable red shift. This behavior is in accordance with an intercalative binding mode, since hypochromism occurs due to π-stacking interactions between aromatic heterocyclic groups and the DNA base pairs, as in the case of classic intercalators (e.g., ethidium bromide) [29]. In addition, the isosbestic point observed at 312 nm (**1**) and 316 nm (**2**) indicated the presence of two species in solution, providing evidence of complex-DNA interaction. These results are similar to those previously reported for various metallointercalators [30,31,32].

In order to quantitatively compare the binding strength of the complexes, the intrinsic binding constants (*K*_b_) of **1** and **2** with CT-DNA were obtained using Equation (1) [28]:

[DNA]/[ε_a_ − ε_f_] = [DNA]/[ε_b_ − ε_f_] + 1/*K*_b_[ε_b_ − ε_f_] (1)

where [DNA] is the concentration of DNA base pairs, ε_a_ is the extinction coefficient of the complex at a given DNA concentration, ε_f_ is the extinction coefficient of the complex in free solution and ε_b_ is the extinction coefficient of the complex when fully bound to DNA. A plot of [DNA]/[ε_a_ − ε_f_] *vs.* [DNA] gives 1/[ε_b_ − ε_f_] as slope and 1/*K*_b_[ε_b_ − ε_f_] as the intercept. *K*_b_ is calculated as the ratio between slope and intercept.

The determined *K*_b_ values were (8.08 ± 0.33) × 10^5^ M^−1^ (compound **1**) and (1.42 ± 0.70) × 10^5^ M^−1^ (compound **2**), indicating that the order of binding affinity was **2** < **1**. The *K*_b_ values of the complexes are in the same order of magnitude of that of ethidium bromide (EB), [*K*_b_ = (1.23 ± 0.07) × 10^5^ M^−1^] [33].

#### 2.5.2. Competitive Binding between EB and Complexes **1** and **2** for CT-DNA

Steady-state competitive binding experiments using complexes **1** and **2**were undertaken to get further proof for the binding of the complexes to DNA. The technique is based on the decrease of EB fluorescence resulting from the competitive displacement of EB from a DNA groove by a compound that competes for the same site.

**Figure 6:**
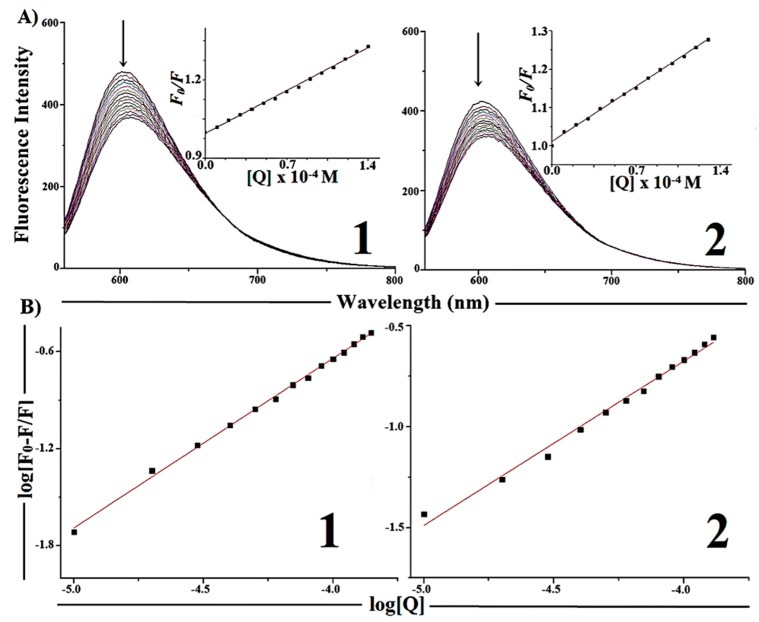
(**A**) Emission spectra of EB-CT-DNA (10 µM Tris-HCl buffer, pH 7.20, T = 25 °C), in the presence of 0, 10, 20, 30, 40, 50, 60, 70, 80, 90, and 100 µM of complexes **1** and **2**. Arrow indicates the changes in the emission intensity with increasing complex concentration. (Inset: Plot of *F_0_*/*F* *vs.* [complex]). (**B**) Plot of log[*F_0_* − *F*/*F*] *vs.* log[Q].

As can be seen in Figure 6, with the increase of complex concentration hypochromism in the emission band at 602 nm (up to 24% for **1** and 20% for **2**) occurs accompanied by a small red shift of the fluorescence band. These effects indicate that the EB molecules are displaced from their DNA binding sites by the complexes under investigation.

The quenching data were analyzed by the Stern-Volmer equation:

*F*_0_/*F* = *K*_sv_[Q] + 1 (2)

where *F*_0_ is the emission intensity in the absence of quencher, *F* is the emission intensity in the presence of quencher, *K*_sv_ is the quenching constant, and [Q] is the quencher concentration. The *K*_sv_ value is obtained as a slope from the plot of *F*_0_/*F* *vs.* [Q] [34].

The apparent binding constants (*K*_app_) were calculated from Equation (3) [35]:

*K*_EB_ [EB] = *K*_app_ [complex] (3)

where *K*_EB_ is 1.0 × 10^7^ M^−1^, [EB] = 10 µM, and [complex] is the complex concentration when the fluorescence intensity of EB is 50%. This value is obtained from the plot *F*_0_/*F* *vs.* [complex] when *F*_0_/*F* = 2.

The binding constants (K) were calculated by the Scatchard Equation (4) (see Figure 6):

log[(*F*_0_ − *F*)/*F*] = log*K* + *n*log[Q] (4)

where *F*_0_ and *F* are the fluorescence intensities of EB in the absence and presence of compound; [Q] is the compound concentration; *K* is the binding constant of compound with EB and n is the number of binding sites. *K* is the antilog of the intercept and n is the slope obtained from the plot of log[(*F_0_* − *F*)/*F*] *vs.* [Q]. Table 2 shows the obtained values of *K*_sv_, *K*_app_ and *K*.

**Table 2:** The Stern-Volmer quenching constant (*K*_sv_), apparent binding constant (*K*_app_), binding constant (*K*) and number of binding sites (n) for the competitive binding between EB bound to CT-DNA and complexes (**1**) and (**2**).

Complexes	*K*_sv_ [M^−1^]	*K*_app_ [M^−1^]	*K* [M^−1^]	*n*
[Cu(H2AcMe)Cl_2_] ( **1**)	(2.35 ± 0.03) × 10^3^	2.34 × 10^5^	(0.35 ± 0.01) × 10^4^	1.04 ± 0.01
[Cu(H2BzMe)Cl_2_] (**2**)	(2.05 ± 0.03) × 10^3^	2.07 × 10^5^	(0.38 ± 0.01) × 10^3^	0.81 ± 0.02

The foregoing results suggest that complexes **1** and **2** are capable of binding DNA in an intercalative way.

### 2.6. Studies of Interactions with Supercoiled Plasmid DNA

**Figure 7:**
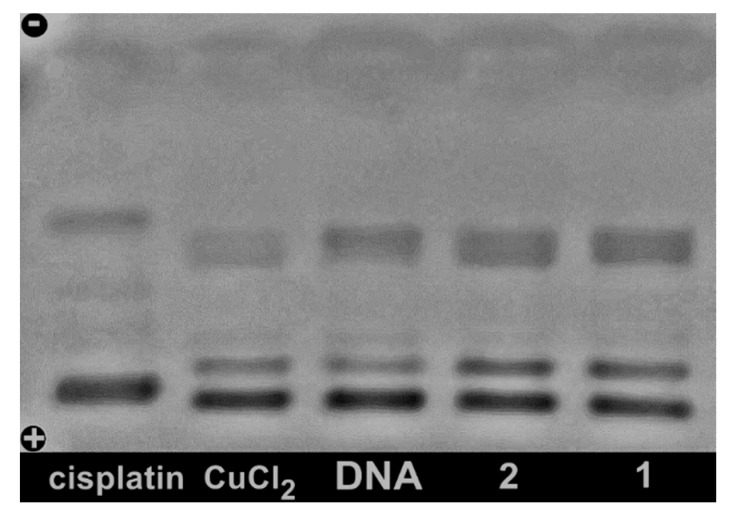
Agarose gel electrophoresis of pUC 19 plasmid DNA treated with selected compounds at 100 μM in Tris-HCl buffer (pH 7.20) incubated at 37 °C for 24 h.

As shown in Figure 7, for complexes **1** and **2** and CuCl_2_ at 100 µM no alteration in the electrophoretic mobility of pUC 19 plasmid DNA was observed in relation to free DNA, whereas strong modification occurs for cisplatin. These results indicate that the complexes under study present a mechanism of interaction with DNA which is different from that of cisplatin. Cisplatin is capable of binding covalently to DNA forming intra-strand crosslinks which cause a decrease in the electrophoretic mobility of all DNA forms. The absence of modifications in the presence of **1** and **2** suggests that both compounds do not form stable adducts with DNA, and hence, they probably do not bind directly to DNA, but rather act as intercalators between the DNA base pairs.

### 2.7. Protein Binding Studies

Fluorescence Quenching of BSA by Complexes **1** and **2**

Anticancer activity may be strongly affected by drug–protein interactions in the bloodstream. Serum albumin, the most abundant and important protein in the plasma, is the major transport protein. Albumin is capable of binding many endogenous and exogenous drugs reversibly. In addition, it may aid in the selective delivery of drugs to the tumor region and facilitate drug access into the cell [36].

Bovine serum album (BSA) is the most extensively studied serum albumin due to its structural homology with human serum albumin (HSA). BSA shows intrinsic fluorescence due to the tryptophan, tyrosine, and phenylalanine residues. Hence, quenching of BSA fluorescence was used to investigate the interaction between BSA and complexes **1** and **2**.

Fluorescence quenching refers to any process that reduces the fluorescence intensity of the fluorophore due to a variety of processes such as excited-state reactions, molecular rearrangement, energy transfer, ground-state complex formation and collisional quenching [37].

**Figure 8:**
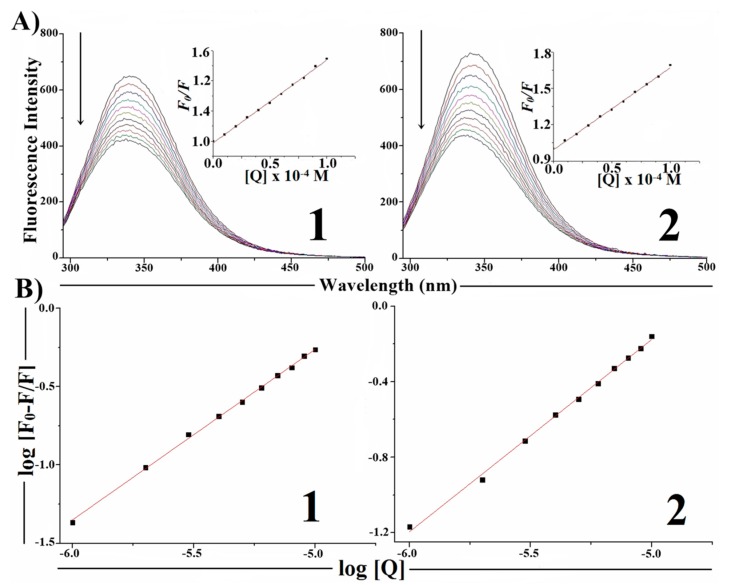
(**A**) Fluorescence emission spectra (excitation at 280 nm) for the **1**-BSA and **2**-BSA systems (phosphate buffer, pH 7.20, T = 25 °C); 1.0 µM of BSA in the presence of 1.0, 2.0, 3.0, 4.0, 5.0, 6.0, 7.0, 8.0, 9.0, and 10.0 µM of compounds **1** and **2**. Inset: plot of *F*_0_/*F* *vs.* [Q]. (**B**) Plot of log [(*F_0_* − *F*)/*F*] *vs.* log [Q] (λ = 345 nm).

Figure 8 shows the effect of increasing the concentration of **1** and **2** on the fluorescence emission of BSA. Addition of the test compounds to BSA resulted in the quenching of fluorescence emission intensity (~35% in **1** and ~40% in **2**) together with a hypsochromic shift of 5–6 nm due to the formation of a complex between the test compounds and BSA.

Based on fluorescence data for λ = 345 nm at 25 °C, the Stern-Volmer quenching constant (*K*_sv_) and the bimolecular quenching rate constant (*k*_q_) were obtained using the classical Stern-Volmer equation (Equation (3)). The value of *k*_q_ was calculated by the *K*_sv_/τ_0_ ratio, where τ_0_ is the average lifetime of the fluorophore in the absence of quencher (τ_0_ =10 ns for BSA) [38].

The *K*_sv_ values for **1**-BSA and **2**-BSA systems were (0.55 ± 0.01) × 10^5^ and (0.68 ± 0.01) × 10^5^ M^−1^, respectively. The calculated values of *k*_q_ for the BSA-complex systems are in the order of 10^12^ M^−1^s^−1^ which are higher than 2.0 × 10^10^ M^−1^s^−1^, the maximum scatter collision quenching constant of quenchers with BSA [38]. This indicates that the fluorescence quenching was not originated by dynamic collision, but it must be caused by a specific interaction between BSA and the complexes, suggesting a static quenching mechanism. Comparable results were obtained from studies of copper(II) complexes with thiosemicarbazones [12].

From the Scatchard equation (Equation (4)) the values of the binding constant (*K*) and number of binding sites (*n*) were calculated (see Figure 8). The *K* and *n* values for the **1**-BSA and **2**-BSA systems were: (1.11 ± 0.04) × 10^5^ M^−1^; 1.06 ± 0.01 and (1.37 ± 0.05) × 10^5^ M^−1^; 1.06 ± 0.01, respectively. From these values it is possible to suggest that complexes **1** and **2** interact with BSA.

**Figure 9:**
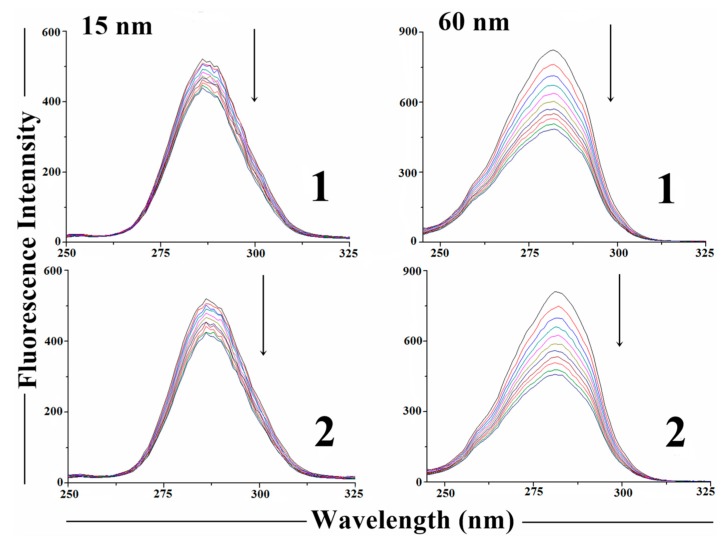
Synchronous spectra of BSA (1.0 µM, phosphate buffer, pH = 7.20) in the absence and in the presence of 1.0, 2.0, 3.0, 4.0, 5.0, 6.0, 7.0, 8.0, 9.0, and 10.0 µM of **1** and **2** at λ = 15 nm and λ = 60 nm, respectively. Arrows indicate that the emission intensity decreases with increasing concentrations of compounds.

Synchronous fluorescence spectroscopy is a very useful method to study the changes in the microenvironment of tyrosine and tryptophan amino acid residues in BSA [39]. The spectra were measured after and before the addition of test compounds. The synchronous fluorescence of BSA is characteristic of tyrosine residues when the difference between excitation and emission wavelength Δλ value is 15 nm and characteristic of tryptophan residues if Δλ value is 60 nm [40,41]. Figure 9 shows the effect of increasing the concentration of compounds on the synchronous spectra of BSA at Δλ = 15 nm and Δλ = 60 nm. In both cases, a decrease in emission intensity of BSA with increasing complex concentration was observed. In the spectra at Δλ = 15 nm, addition of compounds to the BSA solution resulted in a small decrease of fluorescence intensity (~16% for **1** and ~18% for **2**). In the spectra at Δλ = 60 nm, an important decrease of the BSA fluorescence intensity (~41% for **1** and ~44% for **2**) accompanied by a small blue shift of 1nm occurs. These results indicate that the interaction of compounds with BSA affect mainly the conformations of tryptophan residues.

In general, addition of the complexes affects the microenvironment of both tyrosine and tryptophan residues during the binding process and synchronous measurements confirmed the effective binding of the two complexes with BSA. Similar behavior was observed for the interaction between the BSA and copper(II) complexes with other hydrazones [28,42].

In conclusion, our results suggested that complexes **1** and **2** bind to BSA and that this interaction affects the conformations of tyrosine and tryptophan residues, the interaction with tryptophan residues being probably stronger.

## 3. Experimental Section

### 3.1. General Information

All chemicals were purchased from Sigma-Aldrich Brasil (São Paulo, Brazil) and used without further purification. Electronic spectra were acquired with a Shimadzu double beam UV-Vis spectrophotometer UV-2401PC (P/N 206-82201) using 1 cm quartz cells. Measurements of fluorescence were performed on a Varian Cary Eclipse spectrofluorimeter (FL1006m016)-Varian system (Agilent Technologies, Santa Clara, CA, USA) using a 1 cm quartz cell.

### 3.2. Studies of Interactions with DNA

The interaction of complexes **1** and **2** with deoxyribonucleic acid from calf thymus (CT-DNA) was studied in Tris-HCl buffer (NaCl 50 mM, Tris-HCl 5 mM, pH = 7.20). Stock solution of CT-DNA in Tris-HCl buffer was prepared upon shaking in an orbital shaker at 120 rpm at 37 °C for 24 h. Then, the solution was filtered with a Millipore filter (0.45 µm), and its concentration was calculated using the absorption intensity at 260 nm and the molar absorption coefficient (ε) value at this wavelength (6600 M^−1^·cm^−1^) [43]. The stock solutions of the complexes were freshly prepared by first dissolving complexes in DMF and then diluting with Tris-HCl buffer (pH = 7.20). The amount of DMF was kept less than 5% (by volume) for each set of experiments and it presented no effect on any of the experimental results. Absorption titration experiments were performed by increasing the concentration of CT-DNA via successive additions of its stock solution on a fixed concentration of complex. Upon additions in each cuvette (sample and blank) of a same aliquot of CT-DNA (double beam mode), the samples were shaken and left in equilibrium for 2 min, before recording each spectrum.

The affinity of the complexes for DNA was evaluated by a fluorescence technique using ethidium bromide (EB) bound to CT-DNA (EB-CT-DNA) solution in Tris-HCl buffer (pH = 7.20). EB is a planar cationic dye which is widely used as a sensitive fluorescence probe for native DNA. EB emits intense fluorescent light in the presence of DNA due to its strong intercalation between the adjacent DNA base-pairs [44]. The changes in the fluorescence intensity at 602 nm (excitation at 545 nm) of EB-CT-DNA were measured with respect to the concentration of the copper(II) complexes. In fact, when it is removed, EB is non-emissive in Tris-HCl buffer solution (pH = 7.20), due to fluorescence quenching of the free EB by solvent molecules. A competitive binding of the complexes to CT-DNA resulted in displacement of the bound EB, thereby decreasing its emission intensity.

### 3.3. Studies of Interactions with Supercoiled Plasmid DNA

Studies of interactions between the compounds under study and supercoiled plasmid DNA by agarose gel electrophoresis were carried out. Thus, 136 ng of purified plasmid DNA-pUC 19 from *Escherichia coli* (Sigma-Aldrich Brasil, São Paulo, Brazil) were incubated with compounds **1**, **2** and cisplatin at 100 µM in Tris-HCl buffer (NaCl 50 mM, Tris-HCl 5 mM, pH = 7.20). The mixture was incubated at 37 °C for 24 h. Thereafter, the reactions were quenched by adding 5 µL of the loading buffer solution (50 mM Tris, pH 7.20, 0.01% bromophenol blue, 50% glycerol, and 250 mM EDTA). The samples were analyzed by 1% agarose gel electrophoresis in 0.5 × TBE buffer for 1 h 40 min at 75 mV. The gel was stained after electrophoresis in 0.5× TBE buffer with 2.5 µg·mL^−1^ ethidium bromide for 15 min and visualized by UV light.

### 3.4. Albumin Binding Studies

The interaction of complexes **1** and **2** with bovine serum albumin (BSA) was studied in phosphate buffer (8.3 mM, pH = 7.20). Stock solution of BSA (0.5 mM) was prepared in phosphate buffer and stored in the dark in the refrigerator for further use. The stock solutions of complexes were freshly prepared by first dissolving the compounds in DMF and then making the dilutions with phosphate buffer (pH = 7.20). The amount of DMF was kept less than 5% (by volume) for each set of experiment and had no effect on any experimental results.

The binding of the copper(II) complexes to BSA was studied using fluorescence spectra. The emission spectra were recorded in the 295–600 nm range with excitation at 280 nm. The excitation and emission slit widths and scan rates were constantly maintained for all the experiments. BSA solution (1.0 µM) was titrated by successive additions of a stock solution of the complexes (50 mM) using a micropipette. All experiments were performed at 298 K. The synchronous fluorescence spectra of BSA varying the concentration of **1** and **2** were recorded at ∆λ (difference between excitation and emission wavelength) = 15 nm and ∆λ = 60 nm.

If it is assumed that the binding of compounds with BSA occurs at equilibrium, the equilibrium binding constant can be analyzed according to the Scatchard equation [28].

### 3.5. Cell Lines and Culture Conditions

U87 (p53 wild-type glioblastoma multiforme) and T98 (p53 mutant glioblastoma multiforme) malignant human tumor cells and MRC5 (Human Fetal Lung Fibroblast) cells were obtained from the American Type Culture Collection (ATCC, Manassas, VA, USA). Cell lines were grown as monolayer in Dulbecco’s Modified Eagle’s Medium (DMEM, Gibco, Rockville, MD, USA), supplemented with 10% fetal bovine serum (Cultilab, Campinas, São Paulo, Brazil) and antibiotics (50 U/mL penicillin/50 µM streptomycin), in a humidified atmosphere air/CO_2_ (95%/5%) at 37 °C. Cells 80% confluents were used in all experiments. For all experiments, cells were seeded in 96-well plates, at a density of 1000 cells/well. After 24 h incubation cells were treated.

### 3.6. Cytotoxic Activity

To determine the IC_50_ values the cytotoxic effects were quantified using the 3-(4,5-dimethyl-2-thioazolyl)-2,5-diphenyltetrazolium bromide (MTT) colorimetric assay [16]. Briefly, cells were treated with increasing concentrations (10^−12^, 10^−11^, 10^−10^, 10^−9^, 10^−8^, 10^−7^, 10^−6^, 10^−5^, 10^−4^ mol·L^−1^) of either cisplatin (positive control) or test compounds (H2AcMe, H2BzMe, **1** and **2**). The compounds were previously dissolved in dimethyl sulfoxide (DMSO) and the final concentrations were adjusted in DMEM in such manner that the final DMSO concentration was lower than 0.5%. Following 48 h treatment, MTT reagent was added to each well. Following another 4 h of incubation at 37 °C, DMSO was added to each well to dissolve formazan precipitate and absorbance was measured at 570 nm. Tests using DMSO (0.5% in DMEM) as negative control were carried out in parallel. All tests were performed in quadruplicates with full agreement between the results. Statistical analysis was carried out by the unpaired, one-tailed Student’s t testing using a *p* value of 0.05. A value of *p* < 0.05 was considered significant.

### 3.7. Morphological Analysis

Cells were plated in 96-well plates and treated with test compounds (1 × 10^−5^ mol·L^−1^). Morphological changes were analyzed 48 h after the treatment by contrast-phase microscopy (Nikon Eclipse TS100, Tokyo, Japan).

### 3.8. Analysis of Chromosomal Alterations

DAPI (4',6-diamidine-2'-phenindole dihydrocloride) is a fluorescent dye able to bind specifically to double strands of chromosomal DNA [19]. For analysis of chromosomal DNA changes, tumor cells were treated with test compounds at 1 × 10^−5^ mol·L^−1^ for 48 h. Then, cells were washed with phosphate buffer (PBS) and fixed in methanol (70%) for 20 min. Cells were incubated for 30 min with 0.4 μg/mL of DAPI (Sigma). DNA alterations were observed by fluorescence microscopy at 385–410 nm (Nikon, Tokyo, Japan)

### 3.9. Measurement of Reactive Oxygen Species (ROS) Generation

ROS accumulation was detected using dichlorodihydrofluorescein diacetate (DCFH-DA, Sigma). DCFH-DA is a non-fluorescent compound, that when it is taken up by passive diffusion into the cells, is hydrolyzed by esterases to yield non-permeable DCFH. In the presence of ROS, DCFH is oxidized to the fluorescent DCF. Tumor cells were treated with the compounds for 24 h and were then incubated with DCFH-DA (1 × 10^−5^ mol·L^−1^) for 30 min at 37 °C. Following incubation, the cells were washed twice with PBS. Thereafter, the cells were observed under the fluorescent microscope Zeiss Axionplan coupled with a digital camera at excitation and emission wavelengths of 488 and 525 nm, respectively [20]. Data were analyzed using ImageJ (ImageJ, National Institute of Health, Bethesda, MD, USA) and expressed as percentages of DCFDA fluorescence of the corresponding control [45].

### 3.10. Statistical Analysis

The data were expressed as mean + standard deviation (SD) of three independent experiments. One-tailed unpaired Student’s *t*-test was used for significance testing, using a *p* value of 0.05. A value of *p* < 0.05 was considered significant. When appropriate, the statistical significance for multiple comparisons was determined using One Way ANOVA followed by Bonferroni’s test. A value of *p* < 0.05 was considered significant. Prisma Graph Pad for Windows version 5.01 (GraphPad Software Inc., La Jolla, CA, USA) software was used for the statistical analysis.

## 4. Conclusions

Coordination of the hydrazones to Cu(II) resulted in improved cytotoxic activity against U87 cells in both complexes **1** and **2**. The selectivity indexes of both complexes were higher than that of the hydrazone ligands in the two malignant cell lineages.

Preliminary investigation on the mode of action of the studied compounds indicated that both complexes 1 and 2 bind to DNA by an intercalative way. All compounds were able to induce high levels of ROS in the glioma cell lines. Interestingly, H2AcMe and 1 induced lower levels of ROS in MRC5 cells than in U87 glioma cells. Since the cytotoxic effect of complex 1 in wild type p53 U87 cells could be related to its ability to provoke the release of ROS, the cytotoxic effect of 1 on U87 cells might be somehow p53 dependent. No correlation was observed between the cytotoxic effects of the remaining compounds and their ability to provoke the release of ROS. Further studies are needed to clarify the mechanisms of the cytotoxic effects of the compounds under study.
